# Retrospective study of the effectiveness of Intra-Aortic Balloon Occlusion (IABO) for traumatic haemorrhagic shock

**DOI:** 10.1186/1749-7922-10-1

**Published:** 2015-01-06

**Authors:** Takayuki Irahara, Norio Sato, Yuuta Moroe, Reo Fukuda, Yusuke Iwai, Kyoko Unemoto

**Affiliations:** Graduate School of Emergency and Critical Care Medicine, Nippon Medical School, Tokyo, Japan; Department of Primary Care and Emergency Medicine, Kyoto University, Kyoto, Japan; Emergency and Critical Care Center, Nippon Medical School Tama- Nagayama Hospital, Tokyo, Japan

**Keywords:** Trauma, Haemorrhagic shock, Proximal vascular control, Intra-aortic balloon occlusion (IABO)

## Abstract

**Introduction:**

Intra-aortic balloon occlusion (IABO) is useful for proximal vascular control, by clamping the descending aorta, in traumatic haemorrhagic shock. However, there are limited clinical studies regarding its effectiveness. This study aimed at investigating the effectiveness of IABO for traumatic haemorrhagic shock.

**Methods:**

This retrospective, observational study included trauma patients who underwent IABO at the Emergency and Critical Care Center of Nippon Medical School Tama-Nagayama Hospital between January 2009 and March 2013. 14 patients were included to this study who were in shock on arrival (systolic blood pressure [SBP] <90 mmHg or shock index ≥1), underwent IABO for resuscitation and temporary haemostasis, and subsequently underwent haemostatic intervention (operation or transcatheter arterial embolization). Patient characteristics, physiological status, SBP, heart rate (HR), initial fluid and blood transfusion, time course, and total occlusion time were compared before and after IABO as well as between the survived (n = 5) and non-survived (n = 9) groups.

**Results:**

The majority of patients experienced blunt injuries, with an average injury severity score of 29.5. The liver, pelvis, spleen, and mesenterium represented the majority of injured organs. SBP, but not HR, was significantly higher after IABO than before IABO (123.1 vs. 65.5 mmHg, P = 0.0001). The revised trauma score and probability of survival were significantly different between the survived and non-survived groups (both, P = 0.04). The survived group required significantly less blood transfusion volume than the non-survived group (20 vs. 33.7 red blood cell units, P = 0.04). In addition, the survived group required a significantly shorter total occlusion time than the non-survived group (46.2 vs. 224.1 min, P = 0.002).

**Conclusions:**

IABO was used for relatively severe trauma patients. SBP was significantly higher after IABO, but was not related to survival. However, blood transfusion volume and total occlusion time were related to survival; therefore, it is important to reduce or shorten these parameters, i.e., immediate definitive haemostasis. IABO is effective for traumatic haemorrhagic shock; however, it is also important to consider these points and potential complications.

## Introduction

It has been reported that an emergent laparotomy in injured hypotensive patients with massive hemoperitoneum frequently results in cardiac arrest as the abdominal wall tamponade is released. Occlusion of the descending aorta before laparotomy is reportedly necessary for proximal vascular control [1, 2] and can temporarily decrease intra-abdominal bleeding and maintain blood flow to the brain and heart.

Although left thoracotomy with direct clamping of the descending aorta is considered the primary method, it is very invasive, with reported complications such as anterior spinal artery injury or persistent bleeding from intercostal arteries after recovery from shock. In comparison, occlusion of the descending aorta by intra-aortic balloon occlusion (IABO) is less invasive, and the inflation volume and duration can be controlled in response to vital signs. As a result, the latter method is increasingly being used.

IABO, which was developed by Edwards et al. in 1953 [3], was initially intended for surgical treatment of abdominal aortic aneurysms and was later applied to traumatic haemorrhagic shock. It is reportedly effective not only for blunt abdominal injuries but also for retro-peritoneal haemorrhage from a pelvic fracture [4], penetrating abdominal trauma [5], and non-traumatic cases such as post-partum haemorrhage [6]. Stannard et al. described the following IABO steps: (1) arterial access, (2) balloon selection and positioning, (3) balloon inflation, (4) balloon deflation, and (5) sheath removal [7].

The opportunities for IABO use are increasing; however, there have been only a few case reports [6, 8] or experimental studies of animal models (e.g., porcine and dog) [9–14]. Furthermore, there are limited clinical studies regarding its effectiveness. Therefore, this study aimed to retrospectively investigate the effectiveness of IABO for traumatic haemorrhagic shock based on our clinical experiences.

## Materials and methods

### Patients

This retrospective, observational study included trauma patients who underwent IABO at the Emergency and Critical Care Center of Nippon Medical School Tama-Nagayama Hospital between January 2009 and March 2013. Of all trauma patients in this period (n = 540), 21 patients underwent IABO without cardiopulmonary arrest on arrival. Furthermore, 7 patients were excluded if the IABO was inserted as a standby without inflation, inserted preventively for non-shock patients and inflated during haemostatic intervention. The remaining 14 patients were included to this study who were in shock on arrival (systolic blood pressure <90 mmHg or shock index ≥1), underwent IABO for resuscitation and temporary haemostasis, and subsequently achieved haemostatic intervention (operation or transcatheter arterial embolization).

### Indication and procedure

General indication of IABO in our hospital is haemorrhagic shock due to any of the following: (1) intra-abdominal haemorrhage (e.g., liver or splenic injury); (2) retroperitoneal haemorrhage (e.g., renal injury or pelvic fracture); or (3) non-traumatic haemorrhage (e.g., obstetric or gastrointestinal bleeding). In this study, we analysed only trauma patients in case of (1) and/or (2).

During the procedure, generally, the emergency physician inserted the aortic occlusion balloon (Block Balloon™; Senko Medical Instrument Mfg. Co., Ltd., Tokyo, Japan) without radiographic assistance. A 10-Fr sheath was retained in the femoral artery (generally left), the balloon catheter was inserted above the bleeding point and >2 cm below the bifurcation of subclavian artery, and normal saline was injected to inflate the balloon. The procedure was performed with minimum inflation, with monitoring via blood pressure in the upper arm, and minimal occlusion time, achieved by incomplete or intermittent occlusion.

### Data collection

Data were extracted from medical records. The 14 patients were divided into the survived group (n = 5) and non-survived group (n = 9) based on the final recorded outcome. Data regarding the patient characteristics and physiological status in each group were collected. In addition, systolic blood pressure (SBP), heart rate (HR), initial fluid and blood transfusion, time course, and total occlusion time before and after IABO were collected.

Base excess, body temperature, and prothrombin time were collected from the initial data on arrival. Initial fluid and blood transfusion represent the crystalloid volume and red blood cell (RBC) units within 24 hours of arrival, respectively. The injury severity score, revised trauma score (RTS), and probability of survival (Ps) were calculated with commonly used formulas.

### Statistical analysis

Patient characteristics, physiological status, SBP, HR, initial fluid and blood transfusion, time course, and total occlusion time were compared between pre- and post-IABO as well as between the survived group (n = 5) and non-survived group (n = 9) using Wilcoxon signed rank tests and Mann-Whitney *U* tests, respectively. Statistical analyses were conducted using GraphPad Prism 6 (GraphPad Software, Inc., San Diego, CA), and P < 0.05 was considered significant.

## Results

### Patient characteristics

The mean age was 46.9 years old, 71% of the patients were men, and the majority experienced blunt injuries (Table 1).

**Table 1:** **Characteristics of trauma patients who underwent intra-aortic balloon occlusion**

	Values for the entire sample
	(n = 14)
Age (years)	46.9 ± 5.2
Sex (Men:Women)	10:4
Mechanism of injury (Blunt:Stabbing)	13:1
Primary injured organ (n)	
Liver	6
Pelvis	3
Spleen	2
Mesenterium	2
Kidney	1
Femoral artery	1
ISS	29.5 ± 3.6
RTS	5.414 ± 0.308
Ps	0.62 ± 0.09
Location of insertion (n)	
Emergency room	14
Vascular approach	
Right femoral artery	7
Left femoral artery	7

The primary injured organ was defined as the main bleeding organ.

ISS, injury severity score; RTS, revised trauma score; Ps, probability of survival.

Values are mean ± SE.

### Physiological status

Of the measures for physiological status, significant differences were only present between the survived and non-survived groups in the RTS and Ps (both, P = 0.04; Table 2).

**Table 2:** **Physiological status of trauma patients who underwent IABO, based on survival**

	Survived (n = 5)	Non-survived (n = 9)	P value
Age (years)	33.6 ± 4.8	54.3 ± 6.5	0.079
ISS	26.0 ± 6.3	31.4 ± 4.6	0.498
RTS	6.280 ± 0.306	4.933 ± 0.364	0.04
Ps	0.86 ± 0.06	0.48 ± 0.11	0.04
Base excess (mmol/L)	-4.9 ± 2.0	-13 ± 2.9	0.064
Body temperature (°C)	34.9 ± 0.34	35.7 ± 0.31	0.191
Prothrombin time (%)	75.1 ± 11.1	60.5 ± 10.5	0.521

ISS, injury severity score; RTS, revised trauma score; Ps, probability of survival.

Values are mean ± SE.

### Systolic blood pressure and heart rate

**Figure 1:**
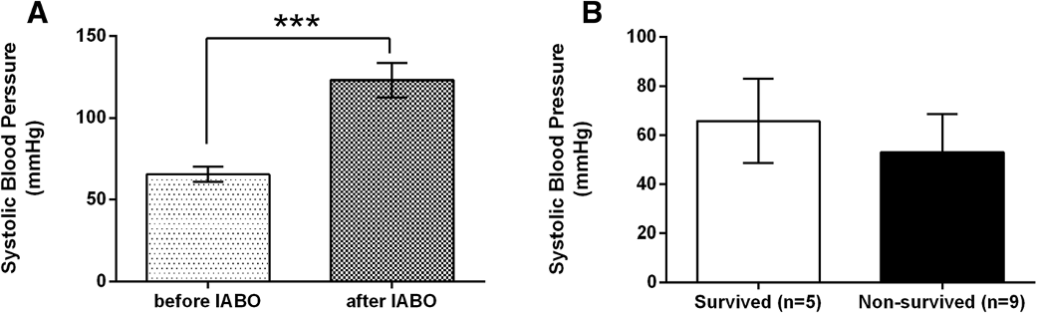
**Comparison of systolic blood pressure (SBP) of trauma patients who underwent intra-aortic balloon occlusion (IABO). A**: Comparison of SBP before and after IABO in all cases (n = 14). **B**: Comparison of the change in SBP (∆SBP) between the survived group (n = 5) and non-survived group (n = 9). Values are reported as mean ± SE, analysed using a Wilcoxon signed rank test **(A)** or Mann-Whitney U test **(B)**. *P < 0.05, **P < 0.01, ***P < 0.001.

SBP was significantly higher after IABO than before IABO, in the entire sample (123.1 ± 10.5 vs. 65.5 ± 4.7 mmHg, P = 0.0001) (Figure 1A). Between the survived and non-survived groups, the change in SBP (∆SBP) was not significantly different (65.8 ± 17.1 vs. 53.1 ± 15.6 mmHg, P = 0.517) (Figure 1B).

**Figure 2:**
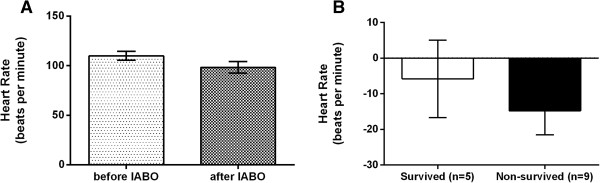
**Comparison of heart rate (HR) of trauma patients who underwent intra-aortic balloon occlusion (IABO). A**: Comparison of HR before and after IABO in all cases (n = 14). **B**: Comparison of the change in HR (∆HR) between the survived group (n = 5) and non-survived group (n = 9). Values are reported as mean ± SE, analysed using a Wilcoxon signed rank test **(A)** or Mann-Whitney U test **(B)**.

The HR after IABO was not significantly different from that before IABO (98.4 ± 5.7 vs. 109.9 ± 4.5 beats per minute [BPM], P = 0.051) (Figure 2A). The change in HR (∆HR) was also not significantly different between the survived and non-survived groups (-5.8 ± 10.9 vs. -14.8 ± 6.7 BPM, P = 0.79) (Figure 2B).

### Initial fluid and blood transfusion

**Figure 3:**
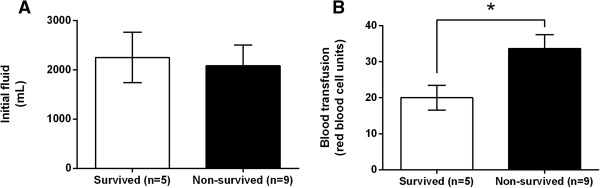
**Comparison of initial fluid and blood transfusion in trauma patients who underwent intra-aortic balloon occlusion (IABO). A**: Comparison of initial fluid (crystalloid volume within 24 hours of arrival) between the survived group (n = 5) and non-survived group (n = 9). **B**: Comparison of blood transfusion (red blood cell [RBC] units within 24 hours of arrival) requirements between the survived group (n = 5) and non-survived group (n = 9). Values are reported as mean ± SE, analysed using a Mann-Whitney *U* test. *P < 0.05.

The initial fluid transition was not significantly different between the survived and non-survived groups (2250 ± 512 vs. 2083 ± 417 mL, P = 0.595) (Figure 3A). However, the survived group required a significantly lower blood volume than the non-survived group (20.0 ± 3.4 vs. 33.7 ± 3.9 RBC units, P = 0.04) (Figure 3B).

### Time course and total occlusion time

**Figure 4:**
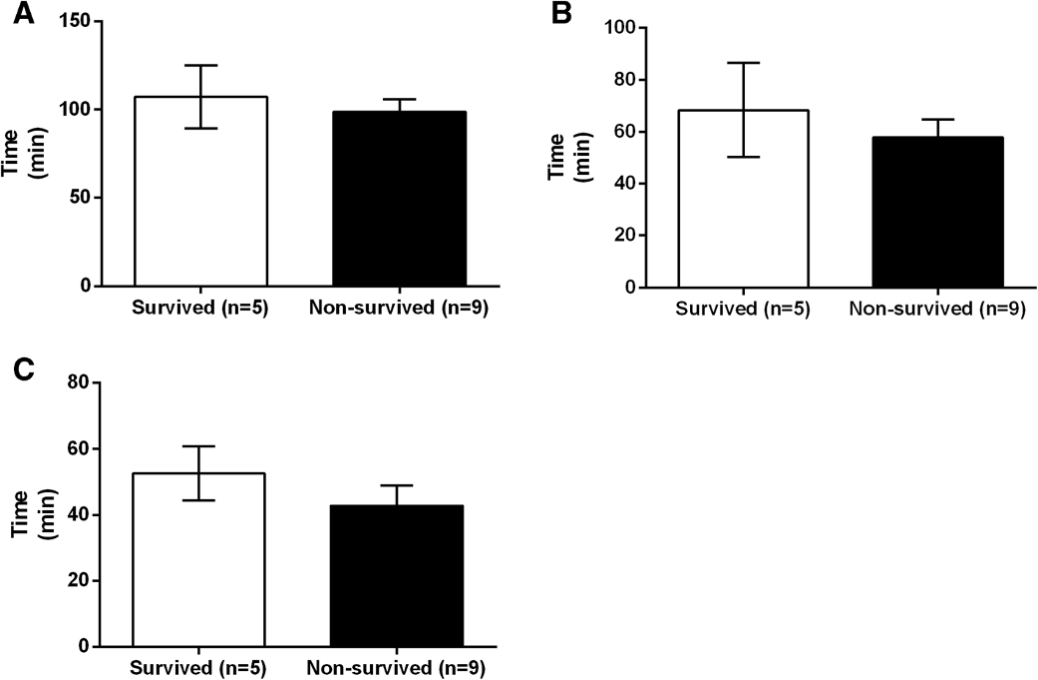
**Comparison of the time course in trauma patients who underwent intra-aortic balloon occlusion (IABO). A**: Comparison of time from injury to IABO insertion between the survived group (n = 5) and non-survived group (n = 9); **B**: Comparison of time from arrival to IABO insertion between the survived group (n = 5) and non-survived group (n = 9); **C**: Comparison of time from IABO insertion to intervention start between the survived group (n = 5) and non-survived group (n = 9). Values are reported as mean ± SE, analysed using a Mann-Whitney *U* test.

**Figure 5:**
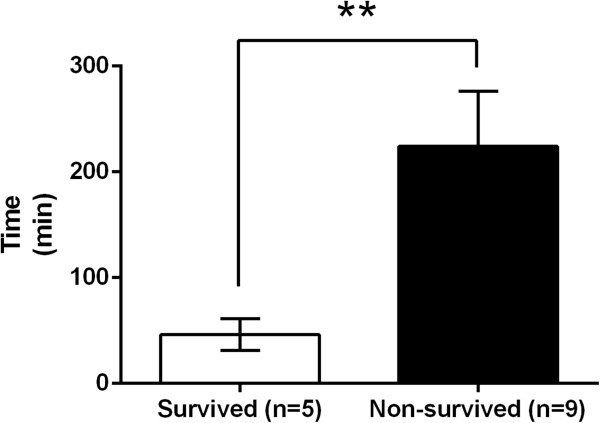
**Comparison of total occlusion time in trauma patients who underwent intra-aortic balloon occlusion (IABO), between the survived group (n = 5) and non-survived group (n = 9).** Values are reported as mean ± SE, analysed using a Mann-Whitney *U* test. *P < 0.05, **P < 0.01, ***P < 0.001.

The comparisons of time course and total occlusion time are shown in Figures 4 and 5.

Between the survived and non-survived groups, there were no significant differences in time from injury to IABO insertion (107.2 ± 17.9 vs. 98.7 ± 7.2 min, P = 0.923) (Figure 4A), time from arrival to IABO insertion (68.4 ± 18.1 vs. 57.9 ± 6.9, P = 0.771) (Figure 4B), or time from IABO insertion to the start of the intervention (52.6 ± 8.2 vs. 42.8 ± 6.3, P = 0.495) (Figure 4C). However, there was a significantly shorter total occlusion time in the survived group than in the non-survived group (46.2 ± 15.0 vs. 224.1 ± 52.1 min, P = 0.002) (Figure 5).

## Discussion

This study demonstrated that IABO was used for relatively severe trauma patients, with an significant increase in SBP after IABO. Similar effects have been shown in other the majority of reports, indicating that IABO is effective for achieving hemodynamic stability. However, based on the significant differences in the blood transfusion volume within 24 hours after arrival between survived and non-survived groups, IABO might only have a temporary haemostatic effect. If definitive haemostasis is not achieved, additional blood transfusion is required, with poorer outcomes.

Therefore, survival depends on lower blood transfusion volumes, by immediate definitive haemostasis, and shorter total occlusion times, by deflating the IABO. It should be noted that poorer outcomes may result from delayed definitive haemostasis, which could occur because of a sense of comfort from the temporary improvement of haemodynamics by IABO. For example, enhanced computed tomography (CT) is often used to search for injury sites, but this could unnecessarily delay definitive haemostasis. Actually we performed enhanced CT after IABO 1 of 5 in survived group and 4 of 9 in non-survived group. Each occlusion time was over 200 minutes in non-survived patients who performed CT. However, the situation may differ by hospital; time is required for the procedure, and IABO has to be deflated temporarily for the injection of contrast medium. As a result, there is a risk that haemodynamics could worsen. Although enhanced CT is necessary when the point of bleeding is unclear and the search for retroperitoneal haemorrhage is unavoidable, the time should be as short as possible.

Physiological status of survived or non-survived patients indicates that IABO was used for relatively more severe trauma patients. Although blood pressure was significantly higher after IABO, it does not appear to be related to survival or have an effect on shock.

Regarding the time course of IABO insertion, it does not appear to be related to survival. Therefore, IABO does not have to be inserted immediately after arrival nor does the intervention need to immediately follow IABO insertion. Instead, the total occlusion time is more important for survival outcomes, as already discussed.

Although patients who were not experiencing shock and underwent IABO for preventive reasons were excluded from this study, we experienced a case of a 46-year-old man with an abdominal stab wound in which IABO was extremely effective for maintaining a good field of operation. His haemodynamics were stable, but IABO was inserted to prevent massive intraoperative bleeding. During the laparotomy, we identified that the stab wound entered the left liver lobe. When the knife was removed, arterial bleeding was observed and controlled by inflation of IABO; as a result, we could complete the liver suture with a good field of view. This effect might be significant for shortening the time to definitive haemostasis. Therefore, we recommend considering IABO for prevention in non-shock cases. For cases that do not present with shock immediately but may experience shock later, it may be best to detain only the sheath initially and be ready to immediately insert an IABO, when necessary.

The major complications of IABO are considered to be aortic injury, dissection, ischemia and reperfusion injury of lower part organs, and thrombosis; therefore, the contraindications include a dissecting aneurysm, significant aortic meandering or calcification, and a bleeding point located above the balloon. On the other hand, it has been reported that complications do not occur with IABO for blunt and penetrating injuries [8]. We also did not experience any aortic injury when we insert IABO blindly not use under radiography. Sovik et al reported that IABO has been used without fluoroscopy in patients with post- partum haemorrhage, and 1 of 6 patients experienced an aortic rupture necessitating surgical repair [6]. Although we also seldom use radiography, it might be helpful to prevent aortic injury. Furthermore we need carefully caution with distal organ ischemia at occlusion point. Markov et al. reported renal dysfunction and liver necrosis have been observed in a swine model at 90 minutes of IABO occlusion; however, this was not related to mortality [12]. In addition, marked splanchnic ischemia during aortic occlusion has been reported in a dog model [14]. We had only one patient with slight renal dysfunction. In this case, the total occlusion time was 37 minutes (incomplete occlusion), and the blood urea nitrogen/creatinine increased to 31.1/1.97 and was improved only by fluid infusion. Although it was not a serious complication, it was likely due to ischemia from IABO; therefore, attention should be paid to this potential complication.

However, limited data from well-organized studies are available, and empirical descriptions indicate that approximately 45 minutes is the limit. It would be helpful to have a staff to manage the balloon and try to minimize incomplete or intermittent occlusion for the maintenance of blood pressure. Moreover, the range of occlusion should be narrowed, e.g., occlusion below the bifurcation of renal arteries in case of pelvic fracture. In addition, the sheath should be as thin as possible. In Japan, a 10-Fr sheath is widely used, but a 7-Fr sheath was recently developed (RESCUE BALLOON®; Tokai Medical Products Inc., Tokyo, Japan) and used clinically.

This study has certain limitations. This study was not a randomized, controlled trial, which may have introduced bias; furthermore, the severity of the patients who survived and who were non-survived was not the same. However, as IABO tends to be used in emergency situations, it is practically difficult to perform a randomized trial. Additional multicentre studies are required to determine the effectiveness of this device.

## Conclusions

Based on our results relating to the effectiveness of IABO for traumatic haemorrhagic shock, a reduction in blood transfusion volume and shorter total occlusion times (i.e., immediate definitive haemostasis) are important for survival. IABO is an effective device to treat traumatic haemorrhagic shock; however, these recommendations and awareness of potential complications are necessary for success.
